# Metabolic imaging distinguishes ovarian cancer subtypes and detects their early and variable responses to treatment

**DOI:** 10.1038/s41388-024-03231-w

**Published:** 2024-12-06

**Authors:** Ming Li Chia, Flaviu Bulat, Adam Gaunt, Susana Ros, Alan J. Wright, Ashley Sawle, Luca Porcu, Maria Vias, James D. Brenton, Kevin M. Brindle

**Affiliations:** 1 Cancer Research UK Cambridge Institute, University of Cambridge, Li Ka Shing Centre, Cambridge, UK; 2 Department of Biochemistry, University of Cambridge, Cambridge, UK; 3 Present Address: Guy’s and St Thomas’s NHS Foundation Trust, St Thomas’ Hospital, London, UK

**Keywords:** Prognostic markers, Cancer imaging

## Abstract

High grade serous ovarian cancer displays two metabolic subtypes; a high OXPHOS subtype that shows increased expression of genes encoding electron transport chain components, increased oxygen consumption, and increased chemosensitivity, and a low OXPHOS subtype that exhibits glycolytic metabolism and is more drug resistant. We show here in patient-derived organoids and in the xenografts obtained by their subcutaneous implantation that the low OXPHOS subtype shows higher lactate dehydrogenase activity and monocarboxylate transporter 4 expression than the high OXPHOS subtype and increased lactate labeling in ^13^C magnetic resonance spectroscopy (MRS) measurements of hyperpolarized [1-^13^C]pyruvate metabolism. There was no difference between the subtypes in PET measurements of 2-deoxy-2-[fluorine-18]fluoro-D-glucose ([^18^F]FDG) uptake. Both metabolic imaging techniques could detect the early response to Carboplatin treatment in drug-sensitive high OXPHOS xenografts and no response in drug-resistant in low OXPHOS xenografts. ^13^C magnetic resonance spectroscopic imaging of hyperpolarized [1-^13^C]pyruvate metabolism has the potential to be used clinically to distinguish low OXPHOS and high OXPHOS tumor deposits in HGSOC patients and to detect their differential responses to treatment.

## Introduction

Ovarian cancer is a leading cause of cancer-related deaths in women [1, 2], with high grade serous ovarian cancer (HGSOC) the most common lethal subtype. Metabolic subtypes have been identified in several cancers [3–6], including HGSOC [7], which have distinct therapeutic vulnerabilities and prognoses. In HGSOC, a high OXPHOS subtype has elevated concentrations of electron transport chain (ETC) components, and increased chemosensitivity, whereas the low OXPHOS subtype exhibits a more glycolytic metabolism and is more drug resistant [7]. Gene copy number signatures have also been used to classify HGSOC, where these can predict relapse following chemotherapy and overall survival based on predicted sensitivity to carboplatin [8]. The mutational processes predicted from these signatures could also affect tumor metabolism and therefore both sets of subtypes could potentially be distinguished using metabolic imaging techniques. These could allow assessment of multiple tumor deposits and thus a more holistic assessment of disease prognosis and selection of the most appropriate treatment.

Positron Emission Tomography (PET) measurements of 2-deoxy-2-[fluorine-18]fluoro-D-glucose ([^18^F]FDG) uptake have been used to assess glycolytic activity in ovarian cancer, however the distinction between benign and malignant lesions can be difficult following ovulation [9] and if there is inflammation or infection [10]. ^13^C magnetic resonance spectroscopic imaging (MRSI) of hyperpolarized [1-^13^C]pyruvate metabolism can also be used to detect increased tumor glycolytic activity [11–14]. Here we investigated whether patient-derived organoids (PDOs) derived from ascites of stage 3–4 HGSOC patients [15], which have different copy number signatures and that can be assigned to the low and high OXPHOS subtypes, display metabolic differences when grown in culture and as xenografts in immunocompromised mice, and whether these differences can be detected using ^13^C MRS measurements of hyperpolarized [1-^13^C]pyruvate metabolism and by [^18^F]FDG PET. We also investigated whether the differential sensitivity of these subtypes to treatment and could be detected using these metabolic imaging techniques.

## Results

### Tumor models

**Fig. 1:**
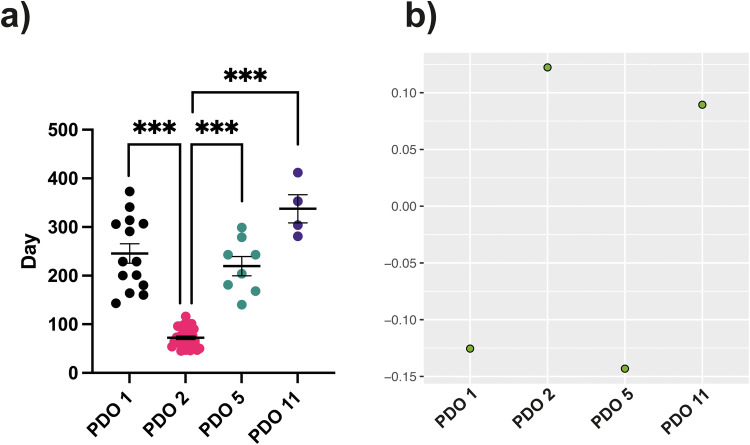
Tumor growth rates and electron transport chain gene set variation analysis. **a** Growth rates of tumors obtained following subcutaneous implantation of PDOs 1, 2, 5, and 11. Time taken for tumors to reach 300 mm^3^. *P* ≤ 0.001***. *P* values were determined using Fisher’s ANOVA. Tukey’s test was used as a post hoc test. **b** Gene Set Variation Analysis (GSVA) of the expression of electron transport chain (ETC) genes.

Tumors were obtained by subcutaneous implantation of organoids derived from patients with stage 3–4 HGSOC (PDOs 1, 2, 5, and 11) [15]. These tumor models have been shown to recapitulate the copy number signatures observed in HGSOC patients, with subcutaneous implantation resulting in the same serous papillary structures as those observed following intrabursal implantation and also reproducing the clinical response to Carboplatin observed in the corresponding patients [16]. Gene Set Variation Analysis (GSVA) of the expression of 96 ETC component genes [7] indicated that PDOs 1 and 5 belonged to a low OXPHOS metabolic subtype, with decreased expression of the ETC genes compared to the entire cohort of genes, and PDOs 2 and 11 to a high OXPHOS subtype, with increased expression (Fig. 1b). Although PDOs 1 and 11 have the highest proportion of copy number signature 1 [15], which clinically is associated with a poorer prognosis [8], tumor growth rate was significantly slower in these two models (Fig. 1a).

### Metabolic imaging

**Fig. 2:**
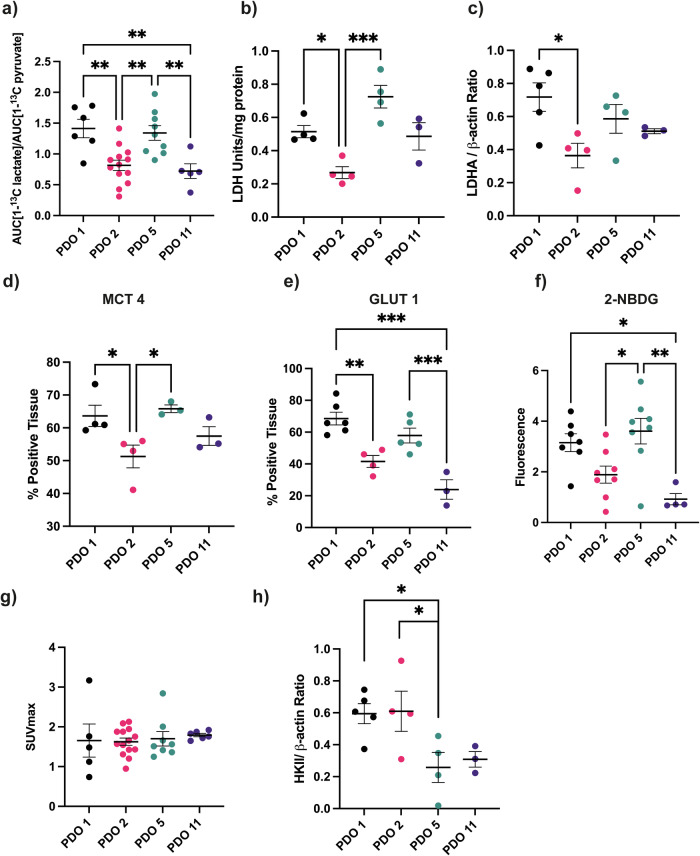
Characterization of tumor metabolism. **a** Tumor lactate labeling following injection of hyperpolarized [1-^13^C]pyruvate. Ratios of the areas under the lactate and pyruvate labeling curves (AUC). *P* < 0.01** (**b**) Lactate dehydrogenase (LDH) activity measured in tumor extracts. *P* = 0.034*, *P* < 0.001***. **c** Expression of lactate dehydrogenase A (LDHA), relative to β-actin, determined by western blot of tumor extracts. *P* = 0.028*. **d** Quantitative analysis of MCT 4 membrane expression in tumor sections. **e** Quantitative analysis of GLUT 1 membrane expression in tumor sections. *P* = 0.003**, *P* < 0.001***. **f** Mean fluorescence intensity of organoids 1 h after incubation with 40 μM 2-NBDG in glucose-free media. Organoids were plated at 150,000/well the day before incubation with 2-NBDG. **g** PET measurements of [^18^F]FDG uptake. Tumor SUVmax at 90 min following i.v. injection of [^18^F]FDG. *P* < 0.05*, *P* = 0.002**. **h** Expression of hexokinase II (HKll) relative to β-actin in tumor extracts. *P* ≤ 0.05*. For (**a**–**f**) *P* values were determined Fisher’s ANOVA. Tukey’s test was used as a post hoc test. For (**g**) *P* values were determined using Welch’s ANOVA. Dunnetts T3 test was used as a post hoc test.

Localized ^13^C MRS measurements of hyperpolarized [1-^13^C]pyruvate metabolism showed that PDO 1 and 5 tumors displayed greater lactate labeling than PDO 2 and 11 tumors (Fig. 2a), and this correlated with LDH activity, which was higher in PDO 1 and 5 tumors compared to PDO 2 tumors (Fig. 2b), and with LDHA expression, which was also significantly higher in PDO 1 when compared to PDO 2 tumors (Fig. 2c). Lactate labeling also correlated with monocarboxylate transporter 4 (MCT4) expression, which was more highly expressed in PDO 1 and 5 tumors when compared to PDO 2 tumors (Fig. 2d) and to a lesser extent with glucose transporter 1 (GLUT1) expression, which was more highly expressed in PDO 1 and 5 tumors (Fig. 2e). Expression of GLUT1 paralleled uptake of the fluorescent glucose analogue, 2-NBDG, in organoids, which was higher in PDO 1 and 5 organoids (Fig. 2f). However, there were no significant differences in tumor [^18^F]FDG uptake (Fig. 2g), which may be explained by contrary differences in hexokinase II (HKII) expression (Fig. 2h), which was higher in PDO 2 than in PDO 5. HKII catalyzes the phosphorylation and trapping of [^18^F]FDG. Representative ^13^C spectra and a [^18^F]FDG-PET image are shown in Fig. S1a, b. All four tumor models showed similar levels of perfusion (Fig. S1c) and tumor volumes at the time of imaging (Fig. S1d–f). A representative western blot of LDHA and HKII expression is shown in Fig. S2a and representative tumor sections stained for MCT4 and GLUT1 expression are shown in Fig. S2b, c. The mouse stromal content of PDO 1, 2, and 5 tumors was less than 15%, although PDO 11 had a higher stromal content of approximately 25% (Fig. S2d, e). Lactate labeling and LDH, MCT4, and GLUT1 expression were not correlated with tumor cell density, which was significantly higher in PDOs 1 and 2 than in 5 and 11 (Fig S2f). The metabolic measurements of increased glycolytic activity in PDOs 1 and 5 are consistent with them belonging to the low OXPHOS metabolic subtype (Fig. 1b).

### c-Myc expression is higher in PDO 1 and EGFR expression in PDO 5

**Fig. 3:**
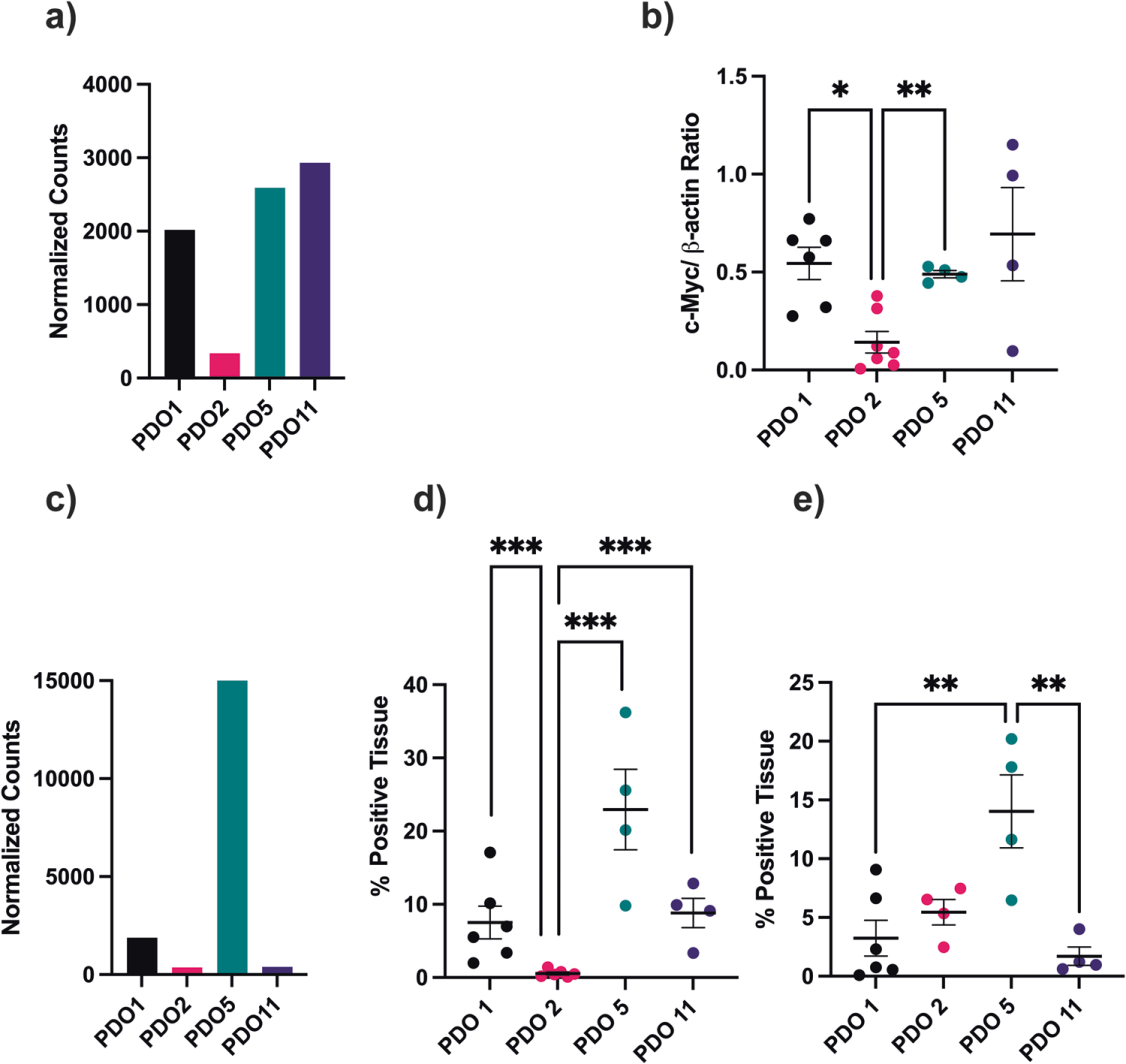
c-Myc shows increased expression in PDO1 and EGFR in PDO5. **a** Normalized counts for c-Myc expression from RNA sequencing data [15]. **b** Densitometric determination of the concentrations of c-Myc relative to β-actin on western blots of tumor extracts. Each biological replicate includes 1–4 technical replicates. *P* = 0.015*, *P* = 0.003**. **c** Normalized counts for EGFR expression from RNA sequencing data [15]. **d** Quantitative analysis of EGFR staining in tumor sections. Percentage of positive stain. *P* = 0.001***. **e** Quantitative analysis of phosphorylated ERK (pERK) staining on tumor sections. Percentage of positive stain. *P* ≤ 0.01**. For (**b**) *P* values were determined using Welch’s ANOVA and Dunnett’s T3 test was used as a post hoc test. For (**d**, **e**) *P* values were determined using Fisher’s ANOVA. Tukey’s test was used as a post hoc test.

One of the most commonly amplified genes in HGSOC is *MYC* [17], which drives the expression of several glycolytic enzymes, including lactate dehydrogenase A (LDHA) and hexokinase II (HKll), and expression of the glucose transporter, GLUT1 [18]. The epidermal growth factor receptor (EGFR), which is overexpressed in up to 60% of ovarian cancers [19], upregulates expression of c-Myc and cyclin D1, promoting both aerobic glycolysis and cell cycle progression [20]. Shallow whole genome sequencing has shown copy number gains for the *EGFR* gene in PDO 5 and the *c-Myc* gene in PDO 1 and analysis of RNA sequencing data showed a correlation between *c-Myc* gene copy number and expression [15]. Analysis of this previously acquired RNA sequencing data showed higher levels of *c-Myc* gene expression in PDOs 1, 5 and 11 as compared to PDO 2 (Fig. 3a). c-Myc protein content in tumor extracts was significantly higher in PDOs 1 and 5 versus PDO 2 (Fig. 3b). A representative western blot is shown in Fig. S3a. EGFR expression was approx. 400-fold higher in PDO 5 and five-fold higher in PDO 1 when compared to PDOs 2 and 11 (Fig. 3c). GSVA showed increased expression of genes in involved in EGFR signalling in cancer (Fig. S3b) in PDO 5. Gene Set Enrichment Analysis (GSEA) for 25 EGFR downstream target genes showed a positive normalized enrichment score for PDO 5 vs PDOs 1, 2 and 11 (Fig. S3c). Staining tumor sections for EGFR showed that expression was highest in PDO 5 (Fig. 3d, Fig. S3d.). EGFR signals to the ERK pathway [21] and staining of tumor sections for phosphorylated ERK (pERK) was higher in PDO 5 tumors (Fig. 3e, Fig S3e).

**Fig. 4:**
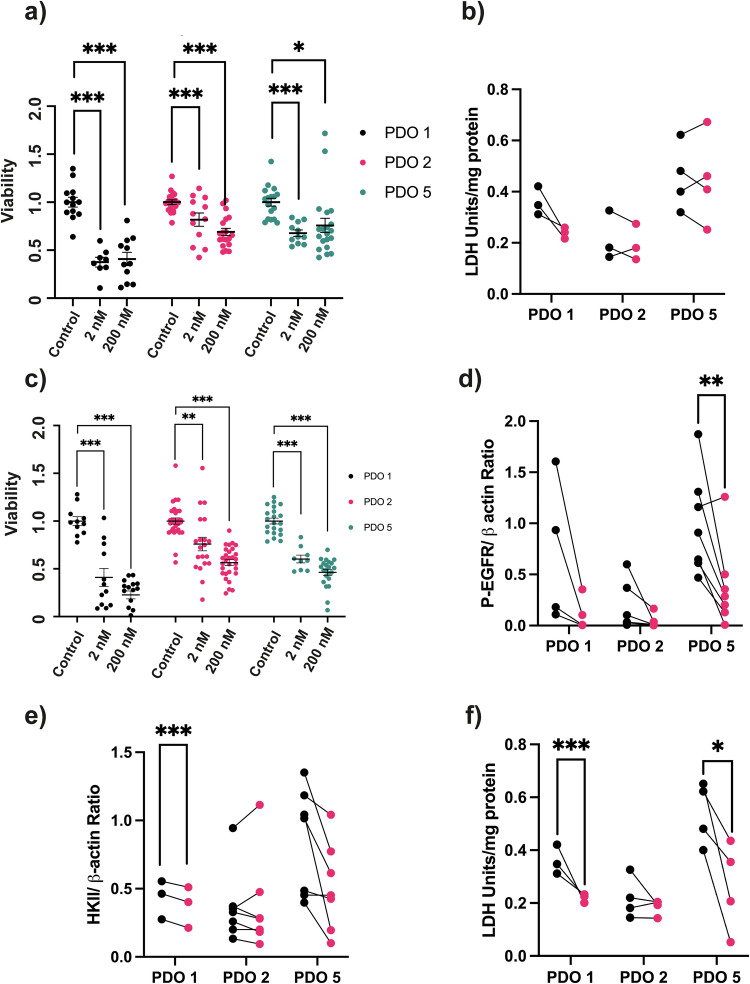
Treatment of organoids with a Myc inhibitor (Myci975). **a** Effect on cell viability. Organoids were plated at 10,000 cells/well and treated for 120 h with 2 nM–200 nM Myci975 and cell viability assessed using the AlarmarBlue assay. Viabilities relative to controls in paired wells are shown. Each biological replicate included 2–4 technical replicates. *P* = 0.022*, *P* < 0.001***. **b** LDH activity following 3 days of treatment with 200 nM Myci975 or matched DMSO control. Each biological replicate included 2–3 technical replicates. Treatment of organoids with an EGFR inhibitor (Erlotinib). **c** Effect of Erlotinib on cell viability. Organoids were plated at 10,000 cells/well and treated for 120 h with 2–200 nM Erlotinib and cell viability assessed using AlarmarBlue. Viability ratios relative to controls in paired wells of the same experiment. Each biological replicate included 2–4 technical replicates. *P* = 0.01**, *P* < 0.001***. For measurements of phosphorylated EGFR (pEGFR), HKII expression and LDH activity organoids were plated at 150,000 cells/well the day before the experiment. Posttreatment measurements were made 3 days after treatment with 200 nM Erlotinib. Each biological replicate included 2–3 technical replicates. **d** P-EGFR expression relative to β-actin. *P* = 0.006**. **e** HKll expression relative to β-actin. *P* < 0.001***. **f** LDH activity. *P* = 0.032*, *P* < 0.001***. A linear mixed-effects model was used. Adjusted p-values of simultaneous z-tests using General Linear Hypotheses are reported.

**Fig. 5:**
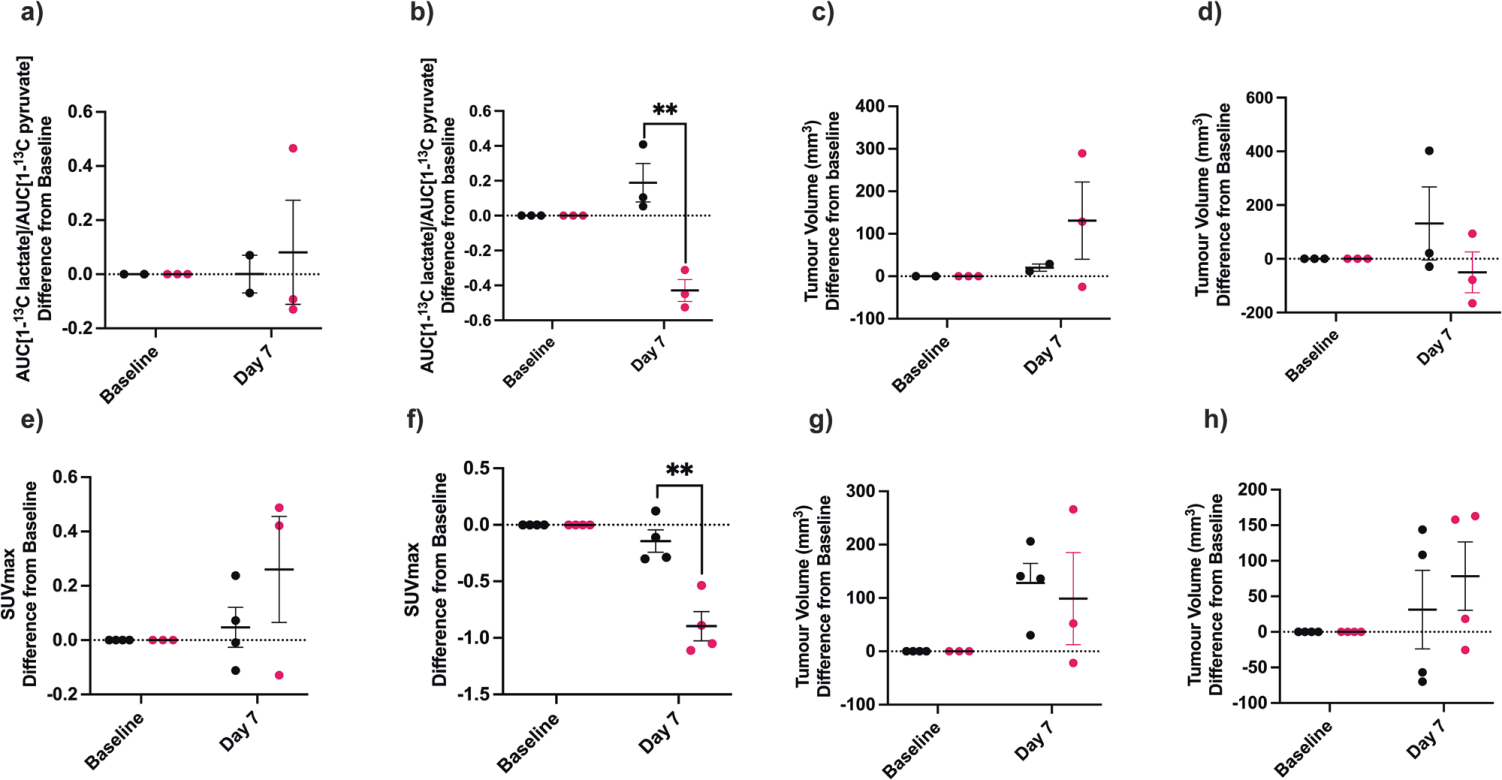
Effect of Erlotinib treatment on tumor lactate labeling and [^18^F]FDG uptake. Effect of Erlotinib treatment on tumor lactate labeling and [^18^F]FDG uptake. Effect of Erlotinib treatment on tumor lactate labeling from hyperpolarized [1-^13^C]pyruvate in (**a**) PDO2 and (**b**) PDO5 tumors. Mice were treated by oral gavage daily for 7 days after baseline imaging on day 0. Control group: 6% Captisol in water, 10 ml/kg/day. Treatment group: Erlotinib 50 mg/kg/day in 6% Captisol. The ratio of the areas under the lactate and pyruvate labeling curves (AUC) were recorded in the same mouse at baseline and after 7 days of treatment and the difference between the 7 day time point and baseline were calculated for Erlotinib-treated and control mice. *P* = 0.008**. Change in tumor volumes (mm^3^) between baseline and after 7 days of treatment in individual treated and control mice with (**c**) PDO2 and (**d**) PDO5 tumors, corresponding to the mice shown in (**a**) and (**b**) respectively. Effect of Erlotinib treatment on [^18^F]FDG uptake in (**e**) PDO 2 and (**f**) PDO 5 tumors. The change in tumor SUVmax between baseline and after 7 days of treatment are shown in individual treated and control mice. *P* = 0.004**. Change in tumor volumes (mm^3^) between baseline and after 7 days of treatment in individual treated and control mice with (**g**) PDO 2 and (**h**) PDO 5 tumors, corresponding to the mice shown in (**e**, **f**) respectively. *P* values were determined using paired Student’s *t* tests.

Treatment of organoids for 5 days with Myci975, a Myc inhibitor that disrupts Myc/Max interaction [22], resulted in a greater decrease in viability in PDO 1 as compared to PDOs 2 and 5 (Fig. 4a) and a decrease in LDH activity in PDO 1 but not in PDO 2 or PDO 5, although this was not signficant (Fig. 4b). Treatment of PDO 1, 2 and 5 organoids for 5 days with Erlotinib, a small molecule inhibitor that blocks EGFR kinase activity [23], resulted in significant decreases in viability in all three models (Fig. 4c) and in the expression of phosphorylated EGFR (P-EGFR), which was significant in PDO 5 (Fig. 4d). HKII expression was decreased in PDO 1 and LDH activity in PDO 1 and 5 but neither were changed in PDO 2 (Fig. 4e, f). A representative western blot of P-EGFR and HKII expression is shown in Fig. S4a. LDHA expression was unchanged in PDOs 1, 2, and 5 following Erlotinib treatment (Fig. S4b). The decreases in HKII and LDH activities in PDO 5, but not in PDO 2, were reflected in metabolic imaging studies on the tumors derived from these organoids. There was a decrease in lactate labeling in PDO 5 but not in PDO 2 tumors (Fig. 5a, b) at 7 days post Erlotinib treatment, at which time there were no changes in tumor volume (Fig. 5c, d). [^18^F]FDG PET showed a significant decrease in SUVmax in PDO 5 tumors but not in PDO 2 tumors (Fig. 5e, f) and again there were no significant changes in tumor volume (Fig. 5g, h). In summary, growth of PDO 1 appears to be driven by c-Myc gene amplification and increased c-Myc expression and PDO 5 by EGFR gene amplification and increased EGFR expression.

### Assessing early responses to standard-of-care treatment

**Fig. 6:**
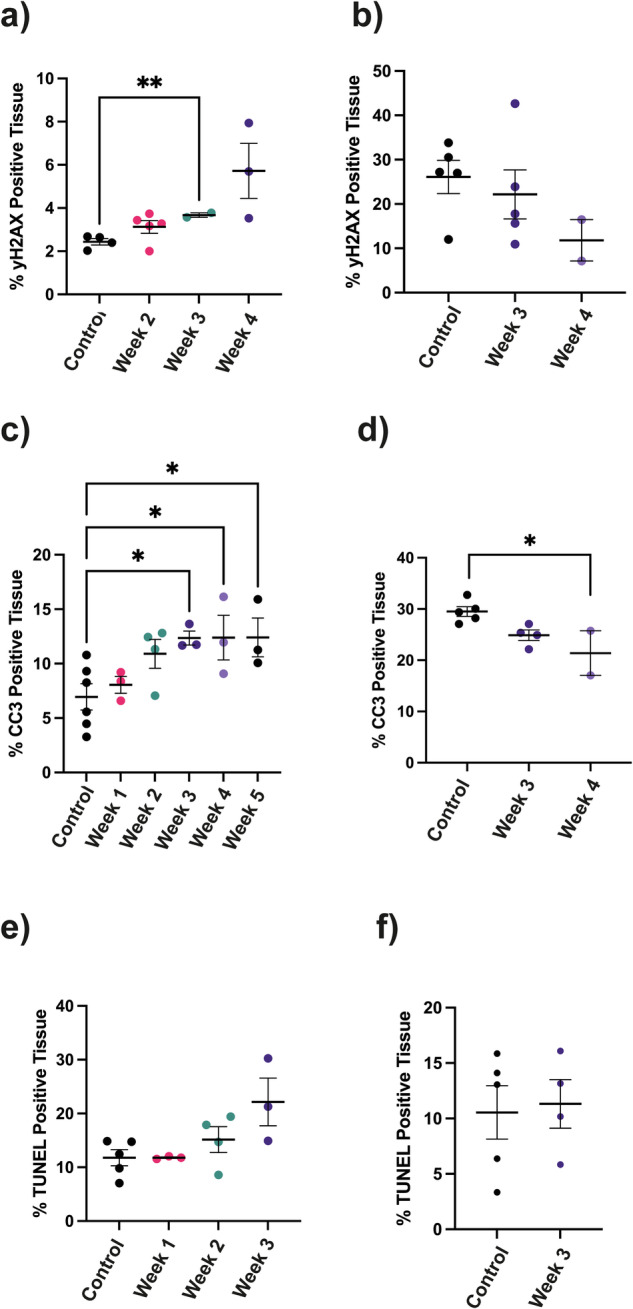
Immunohistochemical analysis of DNA damage and cell death in tumor sections. Mice were treated with 50 mg CBT/kg/week dissolved in mannitol/water for injection (10 mg/ml). Controls were treated with mannitol alone. Times shown are the number of weeks after the start of treatment. **a** γH2AX staining in PDO 2 tumor sections. *P* = 0.006**. **b** γH2AX staining in PDO 5 tumor sections. **c** CC3 staining in PDO 2 tumor sections. *P* < 0.05*. **d** CC3 staining in PDO 5 tumor sections. *P* = 0.02*. **e** TUNEL staining in PDO 2 tumor sections. **f** TUNEL staining in PDO 5 tumor sections. For (**a**, **e**) *P* values were determined using Tamhane-Dunnett Many-to-One Comparison Test. For (**b**–**d**, **f**) *P* values were determined using Dunnett’s many-to-one comparison test on a natural scale.

The standard first-line chemotherapy regimen for ovarian cancer is a combination of Paclitaxel and Carboplatin (CBT) [24]. All 4 models were sensitive to Paclitaxel whereas all the models except PDO 5 were sensitive to Carboplatin and all the models were sensitive to a combination of the two drugs (Fig. S5a–d). Carboplatin treatment results in DNA double strand breaks leading to growth inhibition and cell death [25]. Carboplatin treatment increased phosphorylation of a histone variant, H2AX, which is a marker of DNA double strand breaks (γH2AX) [26], in PDO 2 tumors (Fig. 6a) but not in the Carboplatin resistant PDO 5 tumors (Fig. 6b). Staining for cleaved caspase 3 (CC3), a marker of apoptosis [27], and TUNEL, a marker of DNA damage [28] was increased in PDO 2 tumors but not in PDO 5 tumors (Fig. 6c–f). Representative tumor sections stained for γH2AX, CC3 and TUNEL are shown in Fig. S5e–g.

**Fig. 7:**
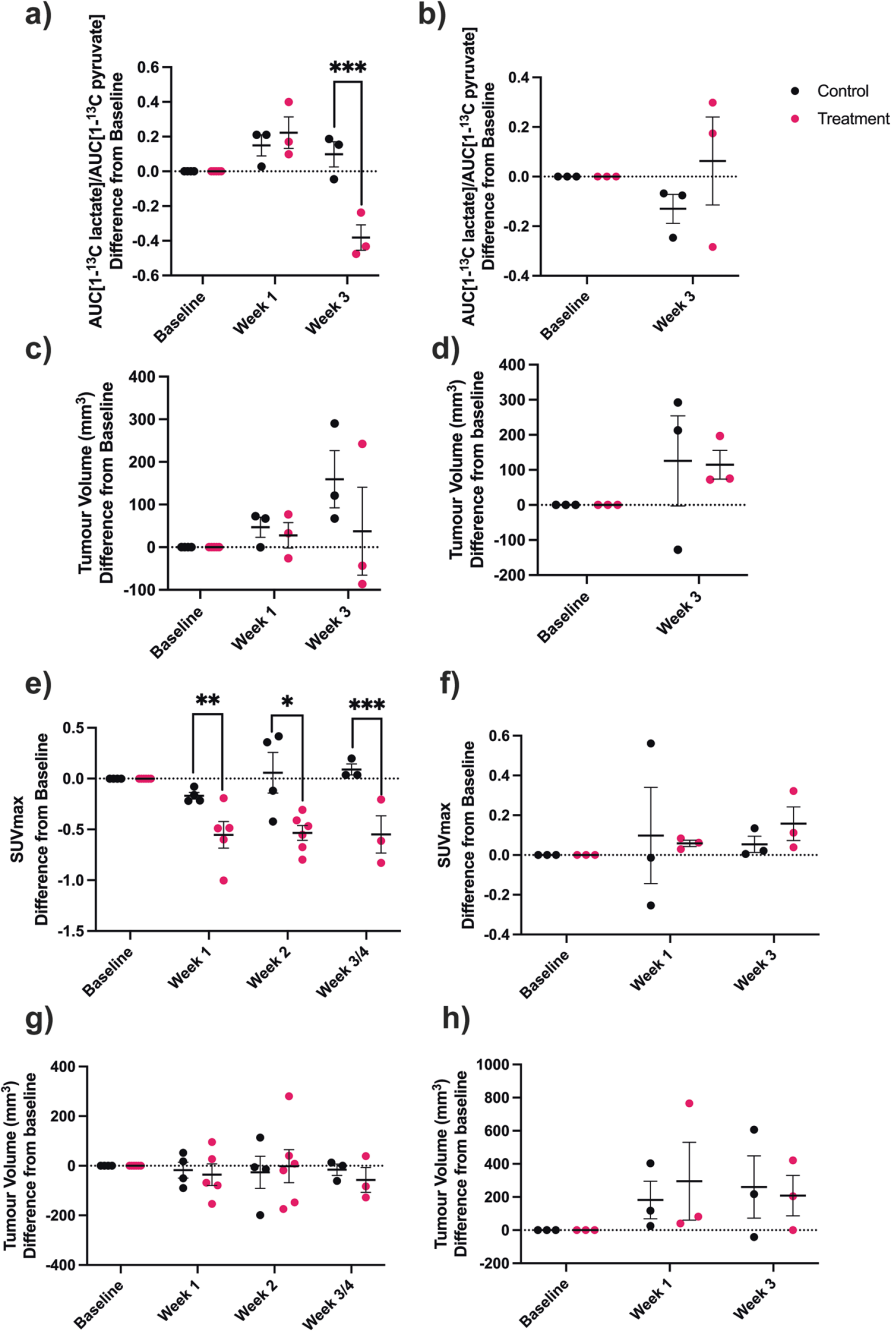
Imaging response to standard-of-care treatment in drug-sensitive and drug-resistant tumors. Imaging response to CBT treatment using hyperpolarized [1-^13^C]pyruvate in (**a**) CBT-sensitive PDO 2 tumors (*P* < 0.001***) and (**b**) CBT-resistant PDO 5 tumors. The ratio of the areas under the lactate and pyruvate labeling curves (AUC) were recorded in the same mouse at baseline and at the indicated times after the start of treatment and the difference calculated for CBT-treated and control mice. **c** Change in PDO 2 tumor volumes (mm^3^) from baseline in individual treated and control mice, corresponding to the mice shown in (**a**). **d** Change in PDO 5 tumor volumes (mm^3^) from baseline in individual treated and control mice, corresponding to the mice shown in (**b**). Imaging response to CBT treatment using [^18^F]FDG-PET in (**e**) CBT-sensitive PDO2 tumors (*P* = 0.015*, *P* = 0.006**, *P* < 0.001***) and (**f**) CBT-resistant PDO 5 tumors. Change in SUVmax from baseline in individual treated and control mice and at the indicated times after the start of treatment. **g** Change in PDO 2 tumor volumes (mm^3^) from baseline in individual treated and control mice, corresponding to the mice shown in (**e**). **h** Change in PDO 5 tumor volumes (mm^3^) from baseline in individual treated and control mice, corresponding to the mice shown in (**f**). For (**a**, **c**, **e**–**h**) *P* values were determined using likelihood ratio tests for maximum likelihood fits for mixed-effects models. Simultaneous Z-tests for General Linear Hypotheses were used as *post hoc* tests. For (**b**, **d**) *P* values were determined using Student’s *t* test.

^13^C MRSI measurements of hyperpolarized [1-^13^C]pyruvate metabolism were compared with PET measurements of [^18^F]FDG uptake in evaluating the early response to Carboplatin treatment in the drug-sensitive PDO2 tumor and the drug-resistant PDO 5 tumor. There was a decrease in lactate labeling in PDO 2 tumors (Fig. 7a) but not in PDO 5 tumors (Fig. 7b), when for both tumor models there were no significant changes in tumor volume (Fig. 7c, d). Similarly, there were decreases in [^18^F]FDG uptake (SUVmax) in PDO 2 tumors (Fig. 7e), but not in PDO 5 tumors (Fig. 7f), again in the absence of changes in tumor volume (Fig. 7g, h).

### Mechanisms responsible for the metabolic response to Carboplatin treatment

**Fig. 8:**
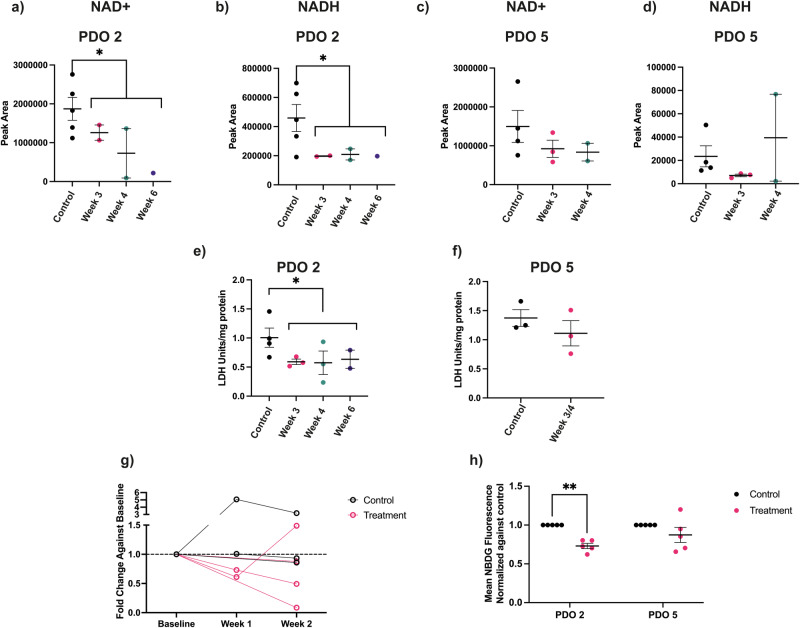
Metabolic changes in PDO 2 and PDO 5 tumors following Carboplatin treatment. Liquid Chromatography - Mass Spectrometry measurements of NAD+ and NADH in tumor extracts before and at the indicated times after the start of treatment. Changes in peak areas show the relative changes in NAD+ and NADH concentrations. Peak areas in extracts of PDO 2 tumors, (**a**) NAD+ (*P* = 0.037*) and (**b**) NADH (*P* = 0.049*) and in PDO 5 tumors, (**c**) NAD+ and (**d**) NADH. LDH activity (units/mg tumor protein) in (**e**) PDO 2 (*P* = 0.024*) and (**f**) PDO 5 tumor extracts before and at the indicated times after the start of treatment. **g** RT-qPCR measurements of GLUT 1 expression in fine needle aspirates from PDO 2 tumors in treated and control mice. Paired values were recorded in the same mouse at baseline and at the indicated times following treatment. The fold change from the baseline value was calculated using the 2(-Delta Delta C(T)) method [1]. **h** Fluorescence measurements of NBDG uptake in organoids at 3 days post treatment with 50 μM Carboplatin. Mean NBDG fluorescence from paired control and treatment organoids were recorded and the data expressed relative to the paired control. *P* = 0.003**. Fisher’s t-test was used for (**a**, **c**–**f**). Welch’s t-test was used for (**b**). *P* = 0.049*. One-sample t- test was used for (**h**).

Following DNA damage, activation of Poly(ADP-ribose) polymerase 1 (PARP1) can deplete the NAD(H) pool [29], which has been shown previously to decrease hyperpolarized ^13^C label exchange between injected pyruvate and endogenous lactate [30]. NAD+ and NADH were depleted in Carboplatin-sensitive PDO 2 tumors (Fig. 8a, b) but not in the resistant PDO 5 tumors (Fig. 8c, d). There was also a significant decrease in LDH activity in PDO 2 tumors (Fig. 8e), but not in PDO 5 tumors (Fig. 8f). There was no significant change in HK activity in either tumor model following treatment (Fig. S6a, b). Fine needle aspiration biopsies showed a general decrease in GLUT1 expression in PDO 2 tumors following treatment (Fig. 8g), consistent with a decrease in 2-NBDG uptake in PDO 2 organoids treated with Carboplatin, which was not observed in PDO 5 organoids (Fig. 8h). There were no significant changes in tumor vascularity in treated PDO 2 tumors (Fig. S7).

## Discussion

Gene copy number signatures have been shown to have prognostic significance in ovarian cancer and have the potential to be used in treatment selection as they discriminate between different mutational processes including homologous recombination deficiency and mitotic abnormalities [8]. However, clinical assessment of these signatures via biopsy is complicated by the presence of multiple tumor deposits that have been shown to differ genomically [31]. Biopsy of all tumor deposits present may not be feasible and moreover there may be heterogeneity even within individual deposits making it difficult if not impossible to discern the genomic signatures present from single biopsies or from liquid biopsies. Similarly, it may be difficult, from biopsies alone, to detect the presence of high OXPHOS tumors, which display higher levels of expression of ETC components and show increased sensitivity to Carboplatin treatment [7]. Here we have investigated whether clinically applicable metabolic imaging methods could be used to discriminate between different gene copy number signatures and between high and low OXPHOS metabolic subtypes in patient-derived ovarian cancer organoids and xenografts and to detect their early responses to treatment, before there were any changes in tumor volume.

Gene copy number signature 1 has been correlated clinically with a poorer prognosis as it is associated with breakage-fusion bridge and mitotic abnormalities that predict reduced sensitivity to carboplatin [8]. However, we observed no correlation here between the proportion of signature 1 and tumor growth rate or with glycolytic activity. PDOs 1 and 11 have much higher proportions of signature 1 [15] and yet formed slower growing tumors when implanted subcutaneously and PDO 2 tumors, which were the fastest growing, showed significantly lower lactate labeling than PDO 1 and 5 tumors. A previous study has also shown no association between OXPHOS subtypes and their global mutation counts or copy number aberrations [7].

The higher lactate labeling from injected hyperpolarized [1-^13^C]pyruvate observed in tumors derived from PDOs 1 and 5 can be explained by their higher LDH activities and increased membrane expression of MCT4, consistent with GSVA of the expression of ETC components, which showed that PDOs 1 and 5 belong to the low OXPHOS metabolic subtype. Increased expression of LDH and MCT4 has been associated with tumor progression [32, 33], including in ovarian cancer [34, 35] and higher lactate labeling from hyperpolarized [1-^13^C]pyruvate has been correlated with LDH and MCT expression in a variety of different tumor types [12].

In contrast to the measurements with hyperpolarized [1-^13^C]pyruvate we observed no significant differences in [^18^F]FDG uptake between the tumor models. Uptake of [^18^F]FDG depends on HK activity and membrane expression of the GLUT glucose transporters and in ovarian cancer patients has been shown to correlate with GLUT1 expression, cell proliferation and tumor grade [36]. While we observed no significant differences in HKII expression between the PDO models there was considerable variation in the membrane expression of GLUT1, which paralleling MCT4 membrane expression was higher in tumors derived from PDOs 1 and 5. However, despite this marked variation in GLUT1 expression we observed no significant differences in [^18^F]FDG uptake and no correlation with tumor growth rate. This has been observed previously in genetically engineered mammary tumor models, where in c-Myc-driven tumors there was no association between [^18^F]FDG uptake and GLUT1 expression or with tumor growth rate or cell proliferation, although there was a correlation with HKII expression [37]. In summary, these studies in preclinical models of HGSOC have shown that imaging with hyperpolarized [1-^13^C]pyruvate has the potential to distinguish between metabolic subtypes in the clinic.

Gene amplification and increased expression of c-Myc in PDO 1 and EGFR in PDO 5 can explain the increased lactate labeling from hyperpolarized [1-^13^C]pyruvate observed in these tumor models. c-Myc has been shown to drive LDHA expression [38] and in glioblastoma patient-derived xenografts was shown to be responsible for increased lactate labeling from hyperpolarized [1-^13^C]pyruvate [39]. Treatment of PDOs 1, 2 and 5 with a Myc inhibitor, Myci975 [22], resulted in decreased viability but this was more marked for PDO 1, in which there was also a significant decrease in LDH activity. EGFR signaling has also been shown to upregulate LDHA expression [40]. Treatment of head and neck squamous cell carcinoma cells (HNSCC) with Cetuximab, an EGFR-blocking monoclonal antibody, resulted in downregulation of HIF-1α, which decreased the expression of LDHA [41], and in patient-derived HNSCC xenografts Cetuximab treatment decreased lactate labeling from hyperpolarized [1-^13^C]pyruvate [42]. Treatment of PDO 1, 2, and 5 organoids with Erlotinib decreased viability, although only PDO 5, which had relatively high levels of EGFR expression and pathway activation, showed a decrease in HKII expression and LDH activity. Erlotinib treatment resulted in a decrease in lactate labeling and [^18^F]FDG uptake in tumors derived from PDO 5, but not in tumors derived from PDO 2 after 7 days of treatment and like the study with Cetuximab and HNSCC xenografts [42] this occurred before there were any significant changes in tumor volume. Although there was a decrease in total LDH activity in PDO 5 there appeared to be no change in LDHA protein expression. This was also observed in Cetuximab-treated patient-derived HNSCC xenografts, where the decrease in lactate labeling was attributed to decreased MCT1 expression, although total LDH activity was not measured in this previous study [42].

Xenografts belonging to the high OXPHOS metabolic subtype have been shown to be sensitive to Carboplatin treatment while those belonging to the low OXPHOS subtype are resistant [7]. The PDO 2 model, a high OXPHOS subtype, was Carboplatin-sensitive in vitro and its response to Carboplatin treatment in vivo could be detected as a decrease in lactate labeling at three weeks after the start of treatment and in [^18^F]FDG uptake as early as one week. These early responses to treatment were detected before there was any change in tumor volume. The decrease in lactate labeling can be explained by a loss of LDH and decreases in NAD+ and NADH concentrations as a result of poly(ADP-ribose) (PAR) polymerase 1 (PARP1) activation [43]. The early decrease in [^18^F]FDG uptake was likely due to a decrease in GLUT1 expression, which was supported by measurements on organoids showing a decrease in 2-NBDG uptake. There were no significant changes in HK activity. A previous study in ovarian cancer xenografts treated with Carboplatin and Paclitaxel showed a decrease in SUVmax by 4 days after treatment, which correlated with a decrease in GLUT gene expression and a significant decrease in HKI but not HKII expression [44]. The PDO 5 model, a low OXPHOS subtype, was Carboplatin resistant and both ^13^C MR measurements of hyperpolarized [1-^13^C]pyruvate metabolism and PET measurements of [^18^F]FDG uptake showed no metabolic changes at up to 3 weeks post treatment, consistent with no significant changes in NAD+ and NADH concentrations, LDH activity, DNA damage or cell death. Drug resistance in this tumor model may be explained by overexpression of EGFR, which has been shown to mediate platinum resistance in ovarian cancer cells [45].

In summary, patient-derived xenografts representative of different HGSOC metabolic subtypes show differences in glycolytic metabolism that can be detected using hyperpolarized [1-^13^C]pyruvate but not by [^18^F]FDG-PET. Myc amplification was identified as the likely mechanism driving increased lactate labeling in the PDO 1 model and EGFR amplification in the PDO 5 model, both of which belong to the low OXPHOS metabolic subtype. However, there was no correlation between the metabolic and growth behavior of these PDO models and their copy number signatures. Both [^18^F]FDG PET and hyperpolarized [1-^13^C]pyruvate could detect response in a high OXPHOS tumor that was sensitive to Carboplatin treatment and non-response in a low OXPHOS tumor that was drug-resistant.

## Materials and methods

### Organoid culture and drug treatment

PDOs were derived from patients with stage 3–4 HGSOC and have been described previously [15]. Cell lines were mycoplasma tested and authenticated using short tandem repeat profiling (STR). Organoids were plated in 96 well plates at 10,000 organoid cells/well and treated, with daily media changes, with drug vehicle (DMSO/water), or 2–200 nM Myci975 (Selleckchem, Catalog No.S8906), 2–200 nM Erlotinib (Selleckchem, Catalog No. S1023), 10 nM Paclitaxel (Selleckchem, Catalog No.S1150) or 50 μM Carboplatin (Selleckchem, Catalog No.S1215). Cell viability was measured after 5 days using AlamarBlue™ Cell Viability Reagent (ThermoFisher). After 4–6 h at 37 °C, fluorescence was measured using a CLARIOStar plate reader (BMG Labtech).

### RNA sequencing analysis

The RNA sequencing data is available at the Gene Expression Omnibus (GEO) under accession number GSE208216 and has been described previously [15]. Analysis of EGFR target gene expression was performed by calculating the Z-score for each target using R software (R Core Team 2022). Z-scores were used to compare RNA normalized counts between the PDO models. Gene sets for EGFR signaling were obtained from the Molecular Signatures Database [46] and from “Signaling by EGFR in Cancer” in the Gene Set Enrichment Analysis Database [47] (https://www.gseamsigdb.org/gsea/msigdb/cards/REACTOME_SIGNALING_BY_EGFR_IN_CANCER) using the R package ‘msigdbr‘ (version 7.5.1). GSVA [48] was used to assess the variation in enrichment of functional gene sets. Enrichment scores were calculated from log2 transformed normalized counts using the GSVA method in R (version 1.50.0) with default settings. Gene Set Enrichment Analysis (GSEA) [49] was carried out using clusterProfiler version 4.6.0 [50] for analysis of the same 25 downstream EGFR targets analyzed with GSVA. To assign PDOs to the high or low OXPHOS subtypes a list of 96 genes associated with ETC components was obtained from [7].

### Flow cytometric measurements of 2-NBD-Glucose uptake

Organoids were plated at 150,000 organoid cells/well on ultra-low attachment plates. PDOs 2 and 5 were treated for 3 days with 50 μM of Carboplatin and then incubated with 40 μM 2-NBD-Glucose (2-NBDG, Abcam, ab146200) in 1 ml of glucose-free DMEM culture medium (A14430-01, Gibco) for 1 h. Uptake was stopped by washing the cells with cold PBS and filtering them through a 35 µm cell strainer (Falcon, Product number 352235) before the addition of 1 μg/ml Propidium Iodide (PI, P4864, Sigma Aldrich). Flow cytometry was performed using a MACSQuant Analyzer 10 (Miltenyi Biotec) for detection of 2-NBDG (excitation 465 nm/emission 540 nm) and PI (excitation 493 nm/emission 632 nm). Data analysis was performed using FlowJo Software (BD Life Sciences, RRID:SCR_008520).

### Generation and treatment of patient-derived xenografts

PDOs 1, 2, 5 and 11 [15] were implanted subcutaneously into the right flank of female NOD SCID gamma (NSG) mice (Charles River Laboratories). Cells (0.5 × 10^6^) were resuspended in 100 μl of PBS and mixed 1:1 with Matrigel (Corning Matrigel Matrix, CLS356230) and then implanted. Animals were not randomized to the different groups and no blinding was used in analysis of the data. Animal experiments were conducted in compliance with licenses issued under the Animals (Scientific Procedures) Act of 1986. Protocols were approved by an Animal Welfare and Ethical Review Body. Tumor sizes were measured using calipers and animals imaged when the tumor volume >0.4 cm^3^.

PDO 2 and PDO 5, tumor-bearing animals were treated by oral gavage daily for 7 days with 50 mg/kg Erlotinib (Selleckchem, Catalog No.S1023) in 6% Captisol (Selleckchem, Catalog No.S4592) and were injected intravenously with 50 mg/kg Carboplatin (Fresenius Kabi; 10 mg/ml dissolved in 10 mg/ml Mannitol (Merck, M4125) in water (B. Braun, SKU: 84/3611655)).

### Reverse Transcription Quantitative Polymerase Chain Reaction (RT-qPCR)

mRNA was extracted from frozen tumor using a RNeasy Mini Kit (Qiagen). cDNA was synthesized using a ProtoScript® First Strand cDNA Synthesis Kit (New England Biolabs). Quantification of glucose transporter 1 (GLUT 1) and glyceraldehyde phosphate dehydrogenase (GAPDH) expression was performed by RT-qPCR using a reaction mixture consisting of 5 μl of PrimeTime Gene Expression Master Mix (Integrated DNA Technologies, catalog number 1055772), 0.5 μl of PrimeTime qPCR Assay mix and 8 ng of cDNA. Reactions were run in a QuantStudio qPCR Thermal Cycler (RRID:SCR_018712) with pre-mixed primers and probes (Integrated DNA Technologies) at 95 °C for 3 min, followed by 95 °C for 5 s, and an annealing temperature of 60 °C for 30 s for 45 cycles. The expression of GLUT 1 was normalized to that of GAPDH. Fold change of gene expression was calculated using the 2(-Delta Delta C(T)) method [51]. Sequences of the primers are given in Supplementary Information.

### Protein extraction

Organoids were plated at 150,000 cells/well and extracted in 80 μl RIPA buffer (Pierce, ref 89900). For tumors, approximately 5 mg of flash-frozen tumor were added to 200 μl RIPA buffer containing complete mini EDTA-free protease inhibitor cocktail (Roche, 04693159001), homogenized using a Precellys Homogenizer (Bertin Instruments) and sonicated using a Bioruptor Plus (Diagenode). Protein quantification was performed using a Direct Detect Spectrometer (Sigma Aldrich).

### Measurements of enzyme activity

Activities are expressed in units per milligram of protein, where 1 unit of lactate dehydrogenase (LDH) is the amount of enzyme that oxidizes 1 μmol of NADH in 1 min at 37 °C. Tumor hexokinase activities were measured using a colorimetric assay kit (ab136957, Abcam), where one unit of hexokinase is defined as the amount of enzyme that will generate 1.0 μmol of NADH per min at pH 8 at room temperature. Absorbances were measured using a CLARIOStar plate reader. Assays were performed with three biological replicates and two technical replicates.

### Western blot

Details of the antibodies are given in Supplementary Information. The secondary antibody was detected using an Amersham Imager 600 (GE Healthcare; RRID:SCR_021853) following incubation with chemiluminescent substrate (SuperSignal™ West Femto Maximum Sensitivity Substrate; Thermo Fisher Scientific, Catalog number: 34094). Densitometry was performed using Fiji ImageJ image analysis software.

### Tumor immunohistochemistry

Tumors were excised and half flash-frozen in liquid nitrogen and the other fixed in formalin for 24 h and then 70% ethanol before paraffin embedding and sectioning into 3 μm thick sections. For fine needle aspiration biopsies, the area around the tumor was shaved and a 23-gauge needle was inserted into the tumor to extract tumor tissue, which was placed on dry ice and stored at −80 °C before RT-qPCR measurements. Analysis was performed as described in [52]. HALO (Indica Labs; RRID:SCR_018350) was used to quantify percentage positivity for each stain. Further details are given in Supplementary Information.

### Liquid chromatography–mass spectrometry measurements of NAD+ and NADH concentrations

Approximately 5 mg of frozen tumor was homogenized in cold solvent (Methanol: Acetonitrile: Water 50:30:20) centrifuged at 21,000 *g* and the supernatant stored at −80 °C. The sample was diluted 5-fold and analyzed using hydrophilic interaction liquid chromatography - high resolution mass spectrometry. Further details are given in Supplementary Information.

### ^13^C Magnetic Resonance Spectroscopy (MRS)

^13^C spectra were acquired at 7 T (Agilent, USA) and analyzed as described previously [53]. [1-^13^C]pyruvate was hyperpolarized using a 3.35-T Oxford Instruments Hypersense polarizer (Oxford Instruments, Abingdon, UK), as described in [53], and also using a home-built polarizer operating at 7 T and 1.2 K [54]. In the latter case, the pyruvate solution was prepared with 25 mM OX063 and in the absence of Dotarem (Gadoteric acid, Guerbet). Dissolution was carried out in 5 mL of D_2_O (with 100 mg/L EDTA) at 160 °C. The dissolved pyruvate was neutralized with 60 mg 10 M NaOH. Hyperpolarized [1-^13^C] pyruvate (0.3 mL, 82 mM, pH 5.0–8.0) was injected via a tail vein catheter and spectra acquired using a slice selective pulse, with a nominal flip angle of 5°, a slice thickness of 8–10 mm, 6010 Hz bandwidth and 1024 data points. The position of the slice was determined from ^1^H images, as described in [53]. Spectra were acquired every second for 3 min. Data were processed in MATLAB (Mathworks, Natick, USA, RRID:SCR_001622) and the ratio of the area under curve (AUC) of the lactate signal to the AUC of the pyruvate signal over 180 spectra was determined.

### [^18^F]FDG PET/CT

Imaging was performed as described in [52]. 2-deoxy-2-[^18^F]fluorodeoxyglucose (15 MBq) (Alliance Medical, Guildford, UK) was injected intravenously via a tail vein. Data were acquired at 90-min post-injection in list-mode format on a NanoPET/CT scanner (Mediso, Hungary). DICOM files were analyzed using Vivoquant 3.0 software (InviCRO, Massachusetts, USA) and the maximum standardized uptake value (SUVmax) and standardized uptake value (SUV) were calculated.

### Statistical analysis

Plots were produced using Prism 9 (RRID:SCR_002798, GraphPad). Diagnostic, model-building and model-fitting were performed using R Statistical Software (v4.4.0; R Core Team 2024). Further details are given in Supplementary Information. A *P* < 0.05 was used as the threshold for statistical significance.

## Supplementary information

Supplementary Information

## Data Availability

The data generated is publicly available in a University of Cambridge Data Repository located at 10.17863/CAM.113603.
