# Supplementary material for: Metabolic imaging distinguishes ovarian cancer subtypes and detects their early and variable responses to treatment
Source: Oncogene. 2024 Dec 6;44(9):563–74. doi: 10.1038/s41388-024-03231-w (PMC11850285; doi:10.1038/s41388-024-03231-w)
Supplement: Supplementary file 1 — Supplementary Information [file 41388_2024_3231_MOESM1_ESM.docx]

# Metabolic imaging distinguishes ovarian cancer subtypes and detects their early and variable responses to treatment

Ming Li Chia^1^, Flaviu Bulat^1^, Adam Gaunt^1^, Susana Ros^1^, Alan J. Wright^1,†^, Ashley Sawle^1^, Luca Porcu^1^, Maria Vias^1^, James Brenton^1^ and Kevin M. Brindle^1,2^

^1^Cancer Research UK Cambridge Institute, University of Cambridge, Li Ka Shing Centre, Robinson Way, Cambridge, UK

^2^Department of Biochemistry, University of Cambridge, Tennis Court Road, Cambridge, United Kingdom

^†^Current address: Guy’s and St Thomas’s NHS Foundation Trust, St Thomas’ Hospital, London, UK

**Supplementary Methods**

*Western Blot*

Protein samples (100 μl) were mixed with 10 μl NuPAGE sample reducing agent (10x, Invitrogen NP0004) and 25 μl LDS Sample Buffer (4x, Invitrogen NP0007). Protein (25 μg) was loaded onto NuPAGE 4-12% bis-Tris gels (Invitrogen) and run at 60V for 30 minutes followed by 120V for 90 minutes in NuPAGE™ 20X MES SDS Running Buffer. Protein was transferred onto nitrocellulose membranes using iBlot2 (Invitrogen) or by wet transfer (Invitrogen™ NuPAGE™ Transfer Buffer 20x, Cat. No. NP00061) overnight at 30V. The membrane was blocked with 3% bovine serum albumin or 5% non-fat dried milk, and then incubated overnight at 4°C with primary antibodies diluted in 3% bovine serum albumin dissolved in Tris-buffered saline (TBS).

The following primary antibodies were used: β-actin (Abcam, mouse monoclonal (ab6276); 1:5000 dilution), anti-EGFR (phospho Y1068) (Abcam, rabbit polyclonal (ab5644); 1:1000 dilution), Lactate dehydrogenase (LDHA) (Cell Signaling Technology, rabbit monoclonal (ab3582);1:2000 dilution), Hexokinase ll (HKll) (Cell Signaling Technology, rabbit monoclonal (ab2867); 1:1000 dilution) and c-Myc (Abcam, rabbit monoclonal (ab32072); 1:1000 dilution).

Following incubation with the primary antibody the membrane was washed 3 times with Tris-buffered saline with 0.1% Tween (TBST) before incubation with an anti-rabbit secondary antibody conjugated to Horseradish peroxidase (HRP, Jackson ImmunoResearch 111-035-14; 1:10000 dilution) or an anti-mouse secondary antibody conjugated to HRP (Jackson ImmunoResearch 115-035-062; 1:10000 dilution) for 1 hour and then washed 3 times with TBST. The secondary antibody was then detected using an Amersham Imager 600 following incubation with a chemiluminescent substrate (SuperSignal™ West Femto Maximum Sensitivity Substrate; Thermo Scientific, Catalog number: 34094). Densitometry was performed using Fiji ImageJ image analysis software and band intensities relative to that for β-actin were calculated.

### *Tumor Immunohistochemistry*

Three μm thick FFPE sections were dewaxed and rehydrated on Leica’s automated ST5020 Multistainer and run on Leica’s Polymer Refine Detection System (DS9800) using the automated Bond-III platform (Leica Biosystems Ltd).

FFPE sections were stained with the following antibodies recognising human proteins:

EGFR (mouse monoclonal, Zymed, 28-0005 1:170 dilution); Monocarboxylate Transporter (MCT4) (rabbit polyclonal, Atlas, HPA021451, 1:170 dilution); Phosphorylated Erk (pERK) (rabbit polyclonal, Cell Signaling Technology, 9101, 1:250 dilution); H2A histone family member X (yH2AX, rabbit monoclonal, Cell Signalling Technology, 9718, 1:200 dilution); Cleaved caspase 3 (CC3, rabbit monoclonal, Cell Signalling Technology, 9664, 1:200 dilution) and TdT-mediated dUTP Nick-End Labeling (TUNEL, DeadEnd Colorimetric system kit Promega Benelux BV).

FFPE sections were also stained with the following antibodies recognising mouse proteins: CD31 (rabbit monoclonal, Cell Signalling Technology, 77699, 1:100 dilution); Anti-smooth muscle actin antibody (ASMA, rabbit polyclonal, Abcam, ab5694, 1:500 dilution); CD45 (rat monoclonal, Abcam, ab25386, 5 μg/mL) and CD68 (rat monoclonal, Abcam, ab53444, 4 μg/mL).

Sodium citrate pre-treatment (Leica’s Epitope Retrieval Solution 1, AR9961) was performed at 100°C for pERK, yH2AX, CD45 and CD68. Tris EDTA (Leica’s Epitope Retrieval Solution 2, AR9640). Pre-treatment was performed at 100°C for 30 minutes for CC3, CD31 and ASMA. Enzyme digestion was performed at 37°C using Leica’s Bond enzyme concentrate (AR9551, 17 mg/mL) for EGFR, MCT4 and TUNEL. Additional protein block (Dako X090930-2) was used for EGFR and DAB Enhancer (Leica, AR9432) was applied for all antibodies.

The anti-mouse and anti-rabbit secondary antibodies are included in Leica’s Polymer Refine Detection System (DS9800). The primary incubation was 15 minutes at room temperature and the secondary an 8-minute incubation. Sections were dehydrated and cleared using Leica’s automated ST5020 multistainer before mounting using Leica’s coverslipper, CV5030 and DPX Mountant (Sigma Aldrich, 06522-500ML). Slides were scanned at 20x magnification with a resolution of 0.5 μm per pixel on an Aperio AT2 (Leica Biosystems). Analysis was performed with HALO software (Indica Labs) to quantify percent positivity for each stain.

For glucose transporter (GLUT1) staining, sections were dewaxed and rehydrated on Leica’s automated ST5020 Multistainer. Sodium citrate pre-treatment was performed at 100°C for 30 minutes before placing in MilliQ water for 30 minutes. The sections were blocked with 5% donkey serum (Sigma Aldrich, D9663) dissolved in TBST for 30 minutes at room temperature. Primary antibody to GLUT1 (Abcam, Ab40084) was diluted 1:200 in 1% donkey serum in TBST and added to the sections for 2 hours at room temperature. Secondary antibody (Alexa Fluor 647 donkey anti-mouse IgG, Invitrogen, A31571) diluted in 1% serum in TBST was added at room temperature for 1 hour. Mounting was performed with ProLong™ Diamond Antifade Mountant with DAPI (ThermoFisher Scientific, P36962). The sections were scanned on an Operetta CLS High Content Analysis System (Perkin Elmer) and analysed using HALO software (Indica Labs) to quantify percent positivity.

For Fluorescence in situ hybridization (FISH) staining, human and mouse centromeres were detected simultaneously on FFPE sections. Three μM sections were baked at 60°C for an hour, deparaffinized in xylene and rehydrated with graded ethanol. Aquarius Tissue Pretreatment Kit (Cat # LPS 100, Cytocell) was used according to manufacturer's instructions. Human Centromere probe (Cat # KBI20000R, Leica Biosystems) and Mouse Pan Centromeric probe (Cat # 1697-MF-01, Cambio) were applied before Fixogum rubber cement (Cat # ICNA11FIXO0125, VWR) was used to seal the coverslips. Slides were treated at 75°C for 5 minutes before hybridization at 37°C overnight. After removing the coverslips, the slides were washed in 0.4X SSC buffer for 2 minutes at 72°C, followed by a brief wash in 2X SSC + 0.05% Tween 20, and then incubated with DAPI counterstain (10µg /mL, Cat # 4083S, Cell Signalling Technologies) at room temperature for 5 minutes. The slides were then washed in water, mounted using Prolong Diamond (Cat # P36970, ThermoFisher Scientific) and imaged on an Akoya PhenoImager HT (Akoya Biosciences). Images were taken at 40x magnification, with a resolution of 0.25 μm per pixel, and analyzed with HALO analysis software.

*Liquid chromatography–mass spectrometry*

The HILIC-HRMS system consisted of a Shimadzu Nexera X2 UHPLC, with Waters Atlantis Premier BEH Z-HILIC VanGuard Fit 1.7 µm 150x2.1 mm column and a Sciex 6600 Triple TOF mass spectrometer. A gradient with decreasing proportion of acetonitrile and increasing proportion of 20 mM Ammonium Carbonate pH 9.4 over 7.8 minutes at 500 µL/min was used to retain and separate the metabolites of interest (NAD+ and NADH), with a further 7 minutes of column re-equilibration, totalling a 15 minute run time.

The method relied on relative quantitation, without calibration standards. The purpose being to measure the relative changes of metabolites between samples, rather than absolute concentrations within a sample. All the samples in each figure were run at the same time and prepared from the same tumour homogenate (100 mg tissue per 1 mL of homogenate). The abundance of a metabolite of interest was measured as peak area from an extracted ion chromatogram.

*Reverse Transcription Quantitative Polymerase Chain Reaction (RT-qPCR)*

The PrimeTime Std qPCR Assay consisted of the following primers for GLUT1:

PrimeTime Primer 1: GGC CAC AAA GCC AAA GAT G
PrimeTime Primer 2: GTG CCA TAC TCA TGA CCA TCG

The primers used for GAPDH:

PrimeTime Primer 1: TGT AGT TGA GGT CAA TGA AGG G

PrimeTime Primer 2: ACA TCG CTC AGA CAC CAT G

PrimeTime Probe with fluorescent reporter, Fluorescein (FAM) was used for both GLUT1 and GAPDH: /56-FAM/AG CTA CCC T/Zen/G GAT GTC CTA TCT GAG C/3IABkFQ/

*Dynamic Contrast Enhanced ^1^H MRI*

Images were acquired at 9.4 T (Bruker) using a 40 mm diameter ^1^H volume coil. A series of T_1_-weighted ^1^H images were acquired for 45 minutes following an injection of 0.2 ml, 20 mM Dotarem (Gadoteric acid, Guerbet) 50 s after the start of image acquisition. Baseline T_1_ measurements were made using an inversion recovery-fast spin echo (FSE) sequence from the same slice. Assuming the R_1_ relaxivity of the contrast agent (Dotarem) to be 2.7 s^-1^ (mmol/L)^−1^, signals were converted on a pixel-by-pixel basis to contrast agent concentration using MATLAB software (RRID:SCR_001622), as described in [1] [2].

*Statistical Methods*

Normal linear regression models were used for hypothesis testing. Fixed effects models were used in Figures 1-3, 5-6, 7b, 7d, 8 and Supplementary Figures S1-2, S5-7. Mixed effects models were used in Figures 4, 7a, 7c, 7e-h and Supplementary Figure S4b. In fixed and random effects models time, experimental groups and their interaction were used as fixed effects. In mixed effects models the random effect was identified by the intercept of the experimental unit. The likelihood ratio test with maximum likelihood as estimator was used to remove potentially useless interaction terms from the models. The assumptions that the underlying residuals are normally distributed and have equal variances (homoscedasticity) were checked using graphical methods (quantile-quantile plots, scatter plots and histograms), descriptive statistics (mean and standard deviation) and formal methods (Shapiro-Wilk test, Bartlett’s test and likelihood ratio test with restricted maximum likelihood as estimator). If the previous methods suggested violation of residuals assumptions the following strategies were applied:

1. data transformation. Data shown in Figures 1a, 3d and S4b were analysed on a log scale while those showed in Figure 3e were analysed on a square root scale. Residuals assumptions appeared to be satisfied on these scales.
2. use of statistical methods allowing unequal variances:

2a. Welch’s *t*-test or ANOVA (Figures 2g, 3b, 6a, 6e, 8b, S1e and S7f).

2b. mixed effects model with unequal variances (Figures 4a, 4c-f and 7e-f).

Otherwise, Student’s *t*-test (Figures 5a-h, 7b, 7d, 8a, 8c-f, 8h, S5a-d, S6a-b and S7e), Fisher’s ANOVA (Figures 1a, 2a-f, 2h, 3d-e, 6b-d, 6f, S1c-d, S1f, S2e-f) and mixed effects models with equal variances (Figures 4b, 7a, 7c, 7g-h and S4b) were used as primary regression models. The likelihood ratio test with maximum likelihood as estimator was used to test primary hypotheses in mixed effects models.

In case of violation of homoscedasticity (heteroscedasticity), Dunnett’s T3 test, Tamhane-Dunnett’s test and simultaneous z-tests for general linear hypotheses based on the joint normal or *t* distribution of the linear function (Figures 4a, 4c-f and 7e) were used as *post hoc* tests. Otherwise, Tukey’s Honestly Significant Difference (HSD) test, Dunnett’s test and simultaneous z-tests for general linear hypotheses (Figures 7a and S4b) were used as *post hoc* tests. The Holm’s sequential Bonferroni procedure was used in Figures S5a-d. Adjusted p-values based on the joint normal or *t* distribution of the linear function (single-step method) were used for simultaneous z-tests.

The following sensitivity analyses were performed to assess the robustness of conclusions of the primary regression model: a) primary regression model on a different scale. Data transformation on natural logarithmic, square root and rank scales were considered; b) primary regression model with unequal variances on the primary scale and on the other scales reported at point a). Generalized least squares were used to fit linear models with unequal variances.

Diagnostic, model-building and model-fitting were performed using R Statistical Software (v4.4.0; R Core Team 2024). Tasks about mixed effects models and linear models using generalized least squares were performed primarily using respectively *lme* and *gls* functions of the *nlme* R package [3], version 3.1.164. The Tukey HSD test was calculated using the *TukeyHSD* function of the *stats* R package, version 4.4.1. Dunnett’s test was calculated using the *DunnettTest* function of the *DescTools* R package, version 0.99.57. Dunnett’s T3 and Tamhane-Dunnett's tests were calculated respectively with the *dunnettT3Test* and *tamhaneDunnettTest* functions of the *PMCMRplus* R package, version 1.9.12. Simultaneous z-tests for general linear hypotheses with the single-step method were calculated using the *glht* function of the *multcomp* R package, version 1.4.26.

**References**

1 Lee H, Mortensen K, Sanggaard S, Koch P, Brunner H, Quistorff B *et al*. Quantitative Gd-DOTA uptake from cerebrospinal fluid into rat brain using 3D VFA-SPGR at 9.4T. *Magn Reson Med* 2018; 79: 1568-1578.

2 Hesse F, Wright AJ, Somai V, Bulat F, Kreis F, Brindle KM. Imaging Glioblastoma Response to Radiotherapy Using 2H Magnetic Resonance Spectroscopy Measurements of Fumarate Metabolism. *Cancer Res* 2022; 82: 3622-3633.

3 Pinheiro J, Bates D. *Mixed-Effects Models in S and S-PLUS*. Springer New York, NY, 2000.

**Supplementary Figures**

**
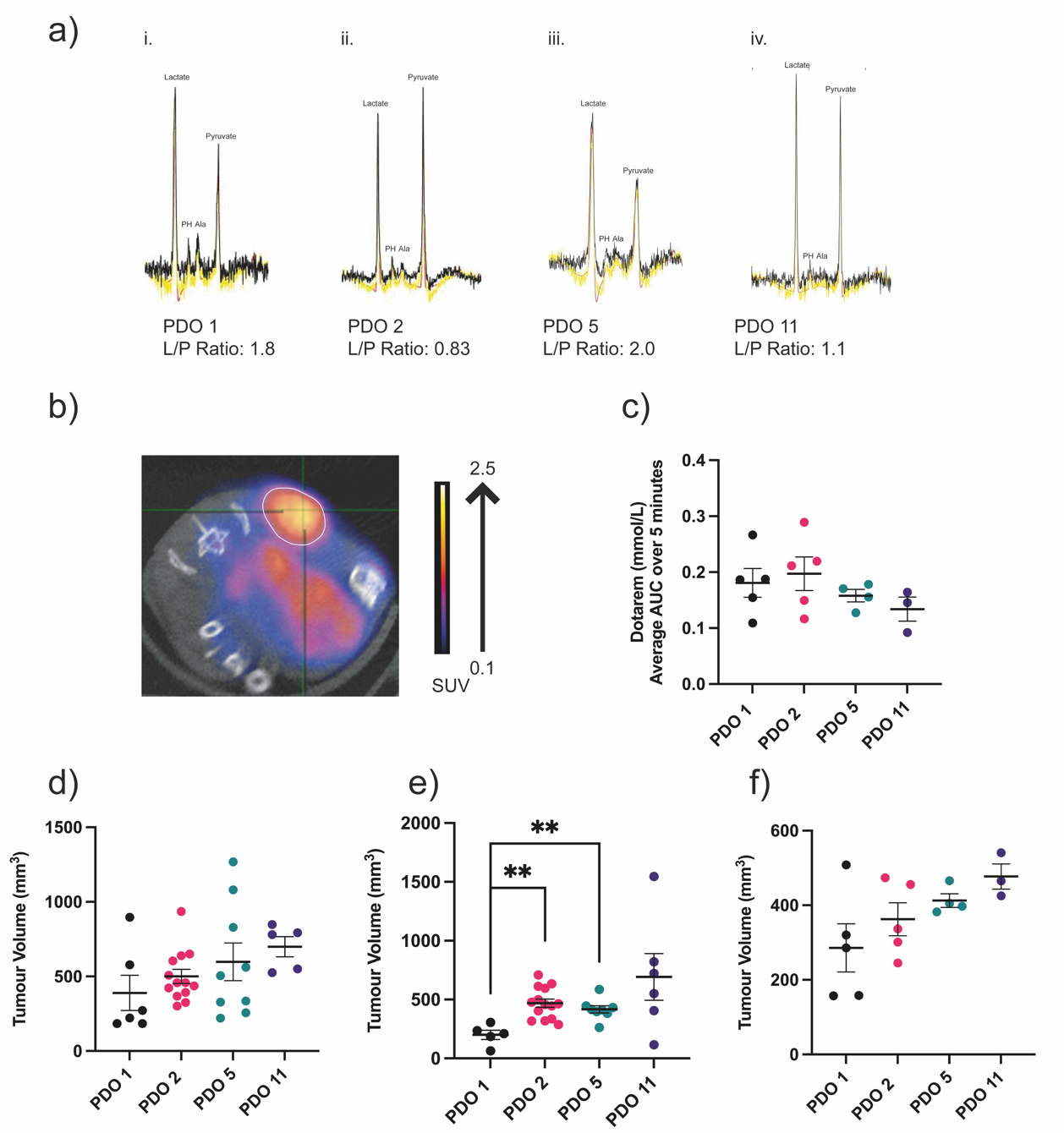
**

**Figure S1.** Imaging tumor metabolism and perfusion. a) Representative tumor ^13^C spectra following injection of hyperpolarized [1-^13^C]pyruvate into animals with i) a PDO1 tumor, ii) a PDO2 tumor, iii) a PDO5 tumor and iv) a PDO11 tumor. Resonances from [1-^13^C]lactate (183 ppm), [1-^13^C]pyruvate (171 ppm), [1-^13^C]pyruvate-hydrate (PH, 180 ppm) and [1-^13^C]alanine (Ala, 177 ppm), are indicated, with the raw spectrum (yellow), model fit (red) and baseline corrected data (black) overlaid. L/P ratio, lactate/pyruvate signal ratio. b) Representative axial [^18^F]FDG PET image from a subcutaneous tumor. c) Average areas under the tumor contrast agent uptake curves (AUC) during the first 5 minutes after contrast agent injection. d) Tumor volumes (mm^3^) at the time the hyperpolarized [1-^13^C]pyruvate experiments were performed. e) Tumor volumes (mm^3^) at the time the PET measurements were performed. P<0.01**. f) Tumor volumes (mm^3^) at the time the perfusion measurements were made. For e) P values were determined using Welch’s ANOVA. Dunnett’s T3 test was used as *post hoc* test. For c), d) and f) P values were determined using Fisher’s ANOVA. Tukey’s test was used as a *post hoc* test. P>0.05.

**
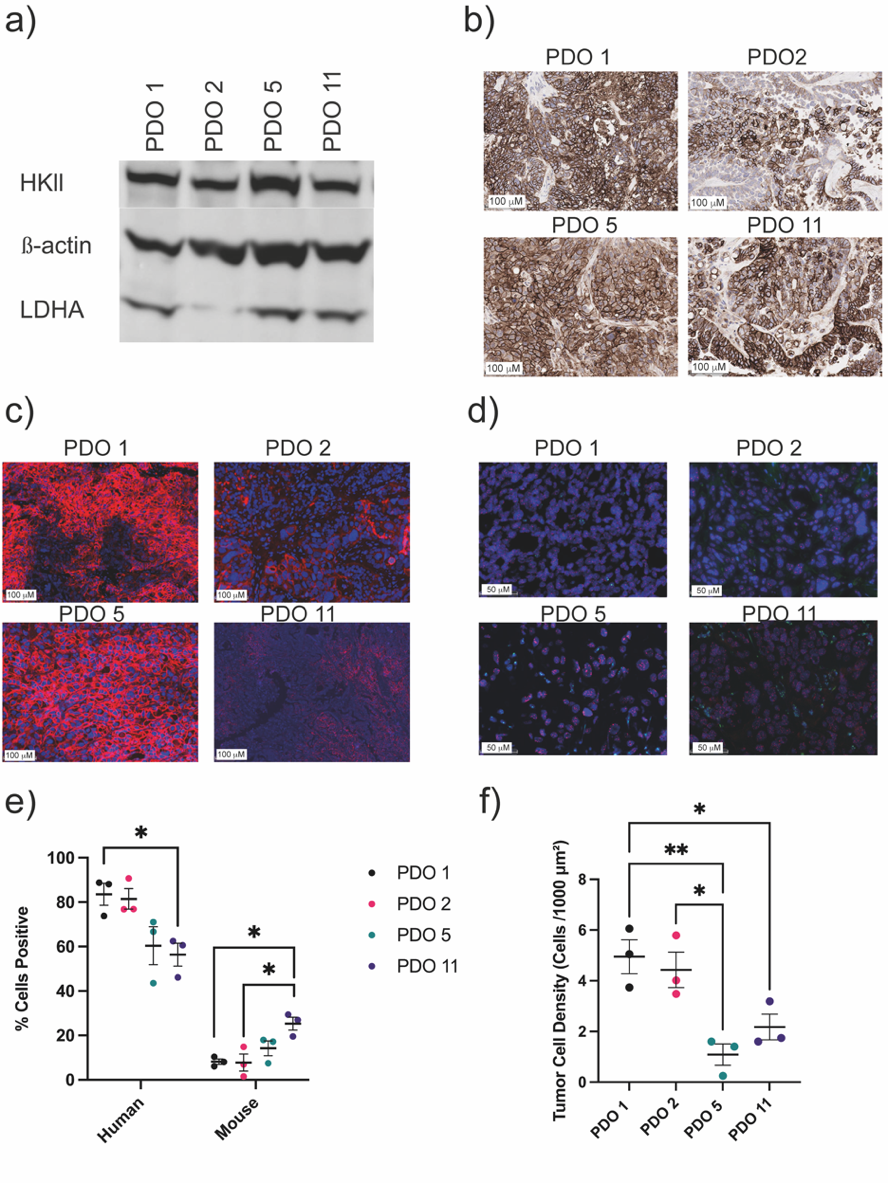
**

**Figure S2**. Characterization of tumor metabolism. a) Representative western blot for lactate dehydrogenase A (LDHA) and hexokinase II (HKll), in 25 μg of protein from a tumor extract. β-Actin was used as a loading control. b) Representative tumor sections stained for MCT4 expression. Images are shown at 20 x magnification and the scale bar is 100 μm. For each independent biological replicate 1-2 sections were stained. c) Representative tumor sections stained for GLUT1 expression. Images are shown at 20 x magnification and the scale bar is 100 μm. d) Fluorescence *in situ* hybridisation of representative tumor sections. Sections were stained with All Human Centromere probe (Cat # KBI20000R, Leica Biosystems, red on tumor sections) and Mouse Pan Centromeric probe (Cat # 1697-MF-01, Cambio, green on tumor sections). The probes were applied to the same slide with a DAPI counterstain. Images were captured at 40x magnification, with a resolution of 0.25 microns per pixel. Images are shown at 40x magnification, and the scale bar is 50 μm. e) Quantitative analysis of the percentage of human and mouse cells in tumor sections. Percentage composition of mouse cells (%). P<0.05*. f) Tumor cell density in the sections shown in (d). P<0.05*, P=0.007**. P-values were determined using Fisher’s ANOVA. Tukey’s test was used as a *post hoc* test.


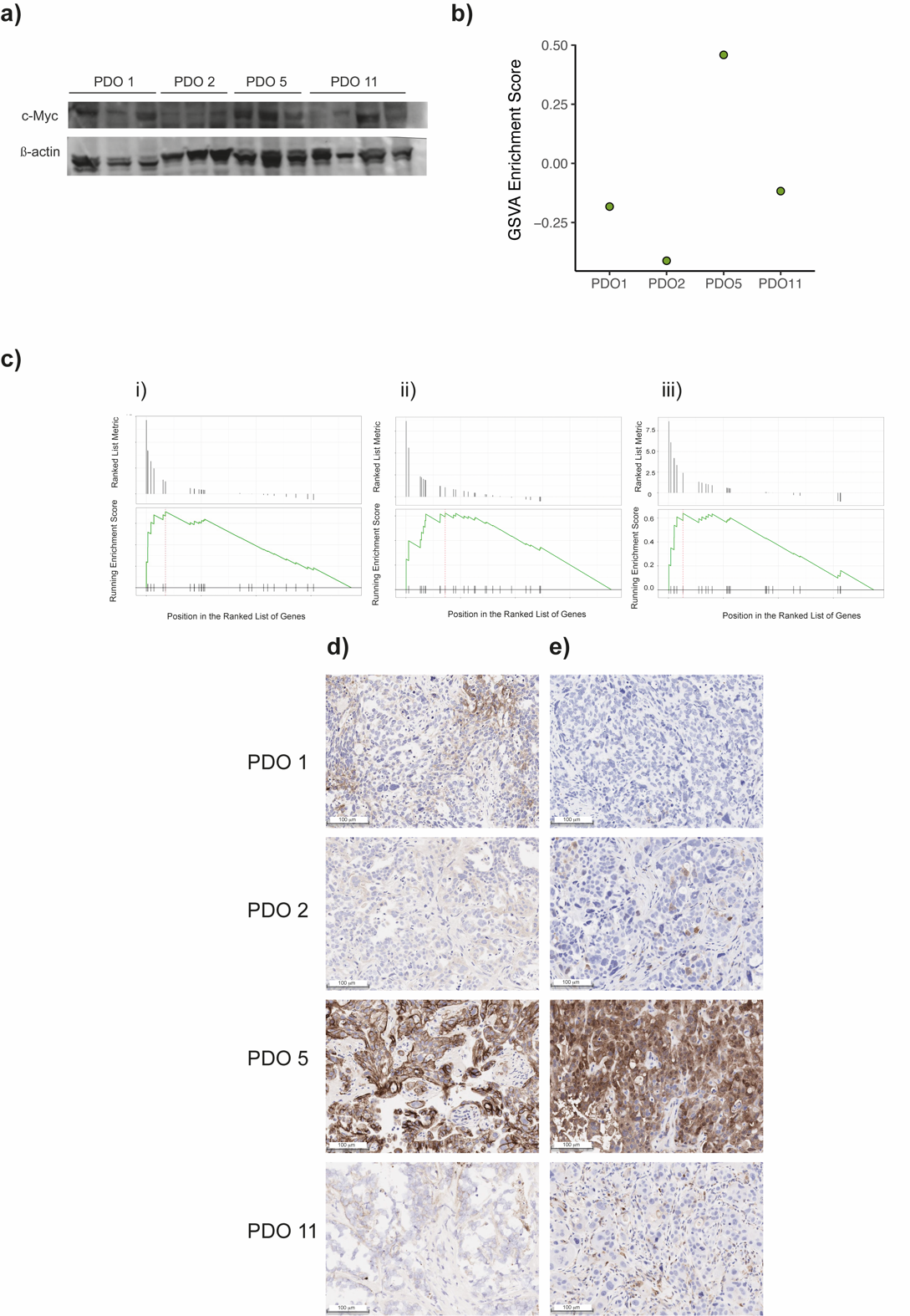


**Figure S3.** a) Representative western blot for c-Myc and β-actin in tumor extracts. b) Gene Set Variation Analysis (GSVA) of functional gene set enrichment. Genes in involved in EGFR signalling in cancer. Same gene set as used for the gene set enrichment analysis (GSEA) analysis shown in c). c) GSEA of EGFR target gene expression. i) PDO 5 vs PDO 1. Normalized Enrichment Score (NES):1.9, False discovery rate (FDR): 0.0012. ii) PDO 5 vs PDO 2. NES:1.8, FDR: 0.031. iii) PDO 5 vs PDO 11. NES: 1.7, FDR: 0.031. Representative tumor sections stained for d) EGFR and e) phosphorylated ERK (pERK). Images are shown at 20x magnification, and the scale bar is 100 μm. For each independent biological replicate 1-2 sections were stained.


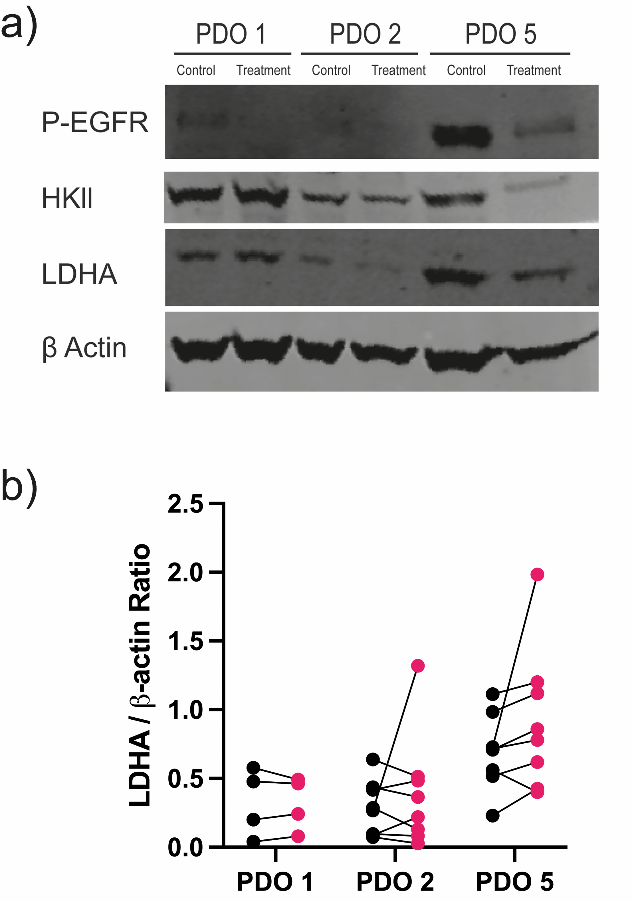


**Figure S4.** a) Treatment of organoid models with an EGFR inhibitor (Erlotinib). Western blot analysis of P-EGFR, HKII and LDHA expression in organoids that had been plated at 150,000 cells/well the day before the experiment and then treated for 3 days with 200 nM Erlotinib or a volume-matched DMSO solvent control. a) Representative western blot using 25 μg protein. The 4 proteins were probed over two membranes, however the protein extract was from the same organoid sample. β-Actin was used as a loading control. b) LDHA expression relative to β-actin. Likelihood ratio test with maximum likelihood as estimator was used to detect primary effect. Simultaneous z test for General Linear Hypotheses was used as a *post hoc* test. P=0.47.


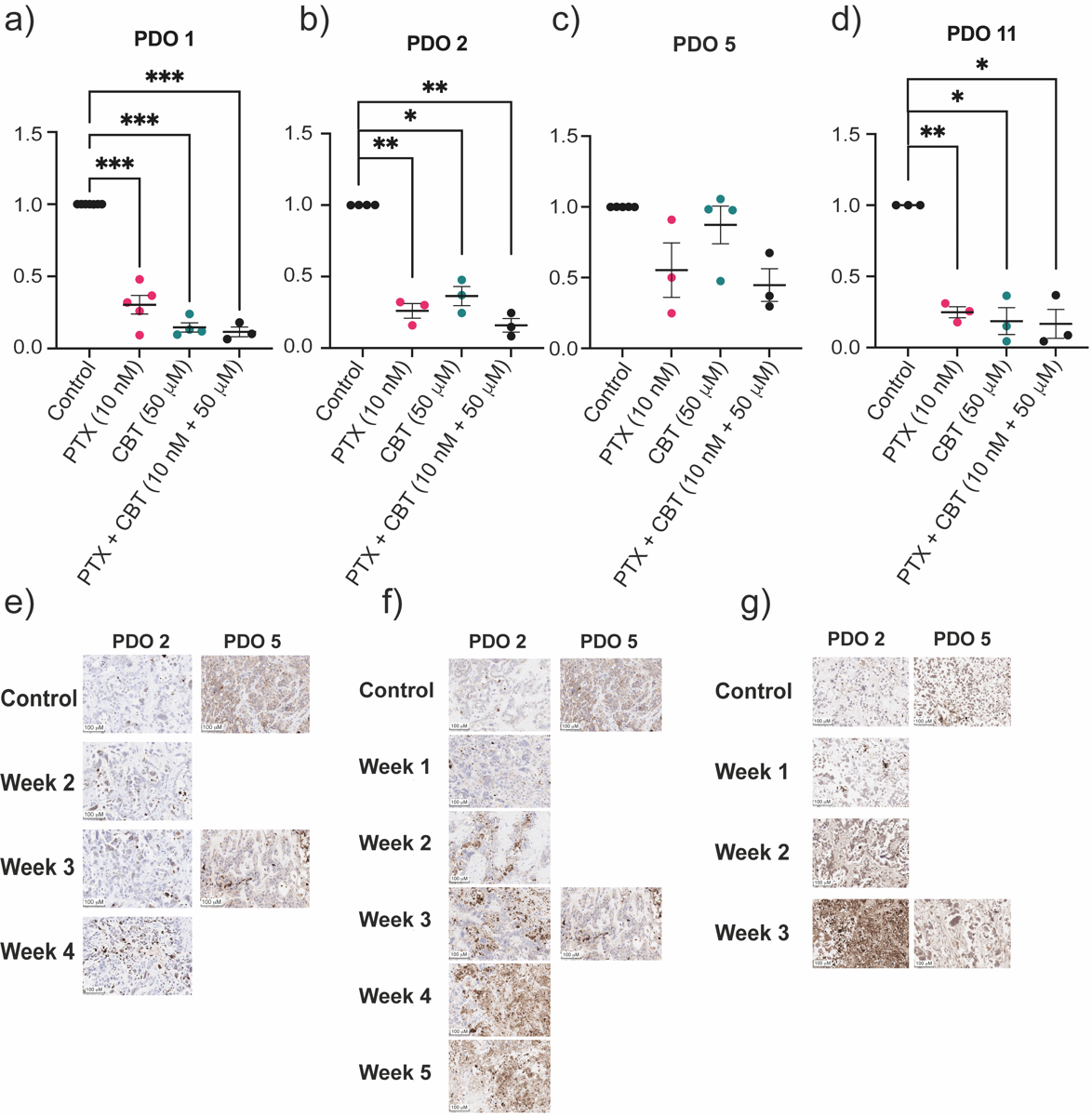


**Figure S5.** Response of drug-sensitive and drug-resistant organoids to standard-of-care treatment. Organoids were plated at 10,000 cells/well and treated for 120 hours with 10 nM Paclitaxel (PTX) or 50 μM Carboplatin (CBT) or a combination of the two. Cell viability was assessed using an AlarmarBlue cell viability assay. Cell viabilities relative to control in paired wells are shown for a) PDO 1, b) PDO 2, c) PDO 5 and d) PDO 11. P-values were determined using a one-sample Student’s t-test. P≤ 0.05*, P≤0.01**, P≤0.001***. Immunohistochemical analysis of DNA damage and cell death in tumor sections. Representative image showing staining for e) γH2AX in PDO 2 and PDO 5 tumor sections, f) CC3 in PDO 2 and PDO 5 tumor sections and g) TUNEL in PDO 2 and PDO 5 tumor sections. Images are shown at 20x magnification, and the scale bar is 100 μm. For each independent biological replicate 1-2 sections were stained.





**Figure S6.** Metabolic changes in PDO 2 and PDO 5 tumors following Carboplatin treatment. Hexokinase activity (mU/mg tumor protein) in extracts of a) PDO 2 and b) PDO 5 tumors at the indicated times after the start of treatment. P values were determined using Fisher’s t-test. P>0.05.


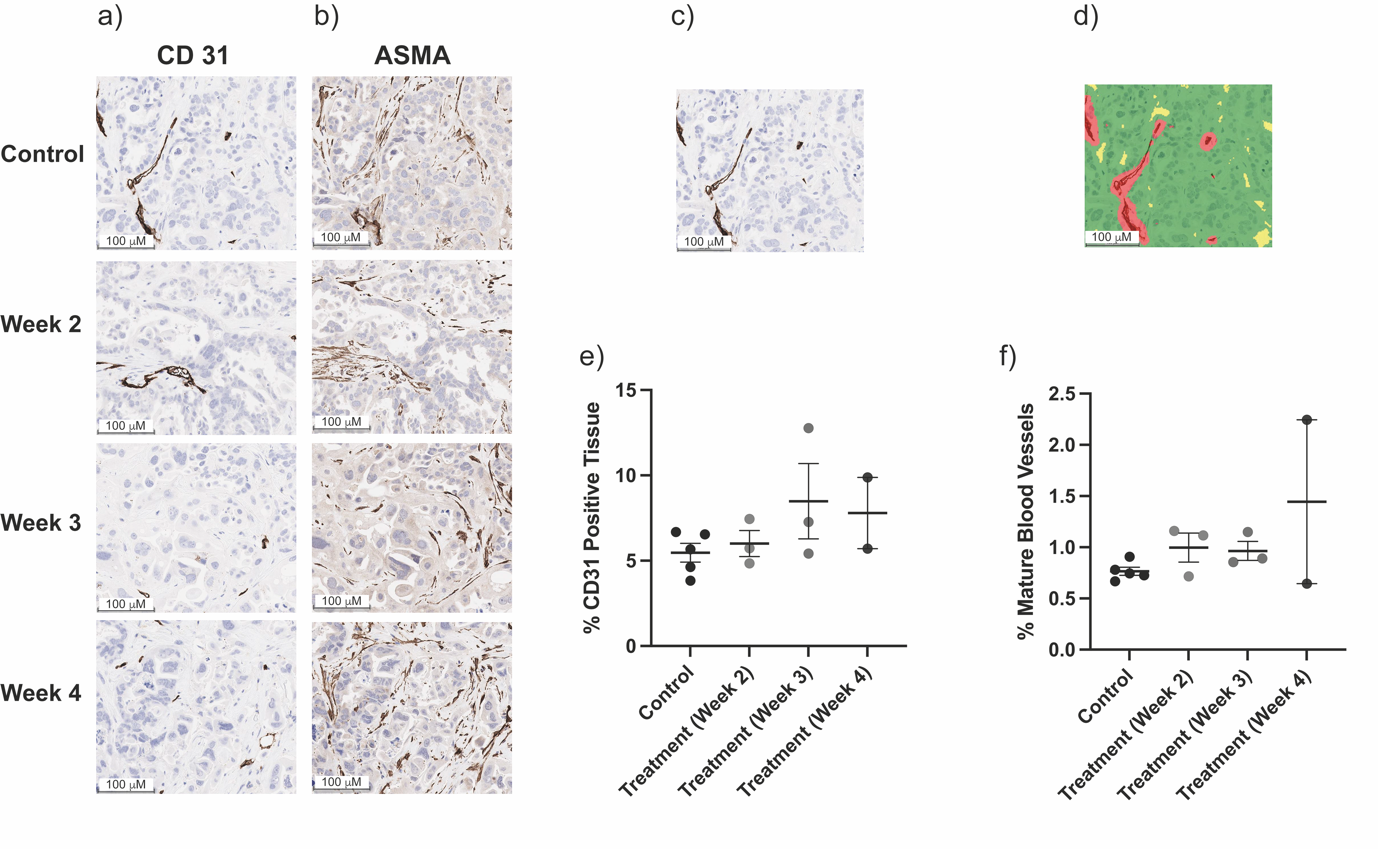


**Figure S7.** Vascularity in PDO 2 tumors following treatment with Carboplatin. Two serial 3 μm sections, one stained for the endothelial cell marker CD31 and one for α smooth muscle actin (ASMA), a marker of mature blood vessels, were co-registered (representative tumor sections are shown). Areas with positive CD31 staining (c, e) were identified and the selected areas classified as CD31 positive (red area in (d)). From the selected red areas, the percentage of cells staining for ASMA (f) were determined. Images were captured at 20x magnification and the scale bar is 100 μm. For e) P values were determined using Fisher’s t-test and for f) using Welch’s t-test. P>0.05.
